# Prognostic histological factors in patients with esophageal squamous cell carcinoma after preoperative chemoradiation followed by surgery

**DOI:** 10.1186/s12885-017-3063-5

**Published:** 2017-01-19

**Authors:** Cheng-Che Tu, Po-Kuei Hsu, Ling-I Chien, Wan-Chen Liu, Chien-Sheng Huang, Chih-Cheng Hsieh, Han-Shui Hsu, Yu-Chung Wu

**Affiliations:** 1 Division of Thoracic Surgery, Department of Surgery, Taipei Veterans General Hospital, Taipei, Taiwan; 2 School of Medicine, National Yang-Ming University, Taipei, Taiwan; 3 Division of Thoracic Surgery, Department of Surgery, Chang Bing Show Chwan Memorial Hospital, Changhua, Taiwan; 4 Department of Nursing, Taipei Veterans General Hospital, Taipei, Taiwan; 5 Division of Thoracic Surgery, Department of Surgery, Taipei-Veterans General Hospital, No. 201, Sec. 2, Shih-Pai Road, Taipei, Taiwan

**Keywords:** Adjuvant therapy, Esophageal cancer, Neoadjuvant therapy, Survival

## Abstract

**Background:**

Pathological response is an important marker for tumor aggressiveness in patients with esophageal squamous cell carcinoma (ESCC) who receive preoperative chemoradiation followed by esophagectomy. We aim to evaluate the prognostic value of histological factors after trimodality treatments.

**Methods:**

91 patients who received preoperative chemoradiation followed by transthoracic esophagectomy between 2009 and 2014 were included. The pathological examination was reviewed. Overall survival and disease free survival were recorded. Survival analysis was performed using the Cox regression model, and the survival curves were compared by the log-rank test.

**Results:**

Survival analysis showed lymphovascular invasion (LVI, hazard ratio [HR]: 2.009, *p* = 0.029), perineural invasion (PNI, HR: 2.226, *p* = 0.019), ypN stage (HR: 2.041, *p* = 0.019), extracapsular invasion (ECI, HR: 2.804, *p* = 0.003), and incomplete resection (HR: 1.897, *p* = 0.039) as unfavorable prognostic factors affecting overall survival (OS). Moreover, tumor regression grade (TRG, HR: 1.834, *p* = 0.038), LVI (HR: 1.975, *p* = 0.038), ECI (HR: 2.836, *p* = 0.003), and incomplete resection (HR: 2.254, *p* = 0.007) adversely affected disease-free survival (DFS). Prognostic classification based on poor primary tumor (TRG2/3, LVI(+), and PNI (+)), lymph node (ypN(+) and ECI(+)), and surgical (incomplete resection) factors significantly predicts OS (*p* = 0.013) and DFS (*p* = 0.017). However, the use of postoperative adjuvant therapy was not a significant prognostic factor even in medium- and high-risk ESCC patients who underwent trimodality treatments.

**Conclusions:**

Histological factors, including primary tumor, lymph node, and surgical factors has high prognostic value for predicting outcomes in ESCC patients receiving preoperative chemoradiation followed by surgery.

## Background

Trimodality treatments, which includes preoperative chemoradiation and surgery, is the approach recommended by the National Comprehensive Cancer Network (NCCN) clinical practice guidelines for most locally advanced esophageal cancers [1]. In the prospective randomized CROSS (Chemoradiotherapy for Oesophageal Cancer Followed by Surgery Study) study, the median overall survival (OS) was 49.4 months in the preoperative chemoradiation plus surgery group vs. 24.0 months in the surgery alone group [2]. However, considerable prognostic differences resulting from varied response to chemoradiation have been observed in patients receiving trimodality treatment. Patients with a partial response to preoperative chemoradiation or no response at all were more likely to have disease recurrence than those with a complete response [3]. Moreover, pathologic non-responders to chemoradiation had no survival benefit when compared with patients who underwent only surgery [4–6]. To evaluate the prognostic impact of pathologic response and provide prognostic discrimination in esophageal cancer patients who received preoperative chemoradiation followed by surgery, a combined classification of primary tumor regression and lymph node status has been recently proposed by Holscher et al. [7] Although patients with a major response (defined by the presence of less than 10% of vital cells in the primary tumor) and ypN0 had a 5-year survival rate of 64%, the rate was only 18% in those with a minor response and ypN(+).

However, Holscher’s prognostic classification was based on histological response in esophageal adenocarcinoma. Information regarding the prognostic impact of histological response in esophageal squamous cell carcinoma (ESCC) is limited in the literature. In this study, we aimed to evaluate the prognostic impact of histological factors in patients with ESCC after preoperative chemoradiation and esophagectomy.

## Methods

We retrospectively reviewed the records of 91 consecutive ESCC patients who had undergone preoperative chemoradiation followed by transthoracic esophagectomy at the Taipei Veterans General Hospital between January 2009 and December 2014. Since patients with cervical ESCC would be treated with definitive chemoradiation, only patients with intrathoracic ESCC were included. Preoperative staging workup were previously described [8]. In particular, endoscopic ultrasound (EUS) was an optional procedure, but was required for confirmation of cT1 or cT2 lesions.

Preoperative chemoradiation included two courses of chemotherapy that were administered with a 4-week interval. The chemotherapy regimen included 80 mg/m^2^ of cisplatin administered intravenously on day 1 followed by continuous intravenous infusion of 600 mg/m^2^ 5-fluorouracil (5-FU) and 90 mg/m^2^ leucovorin on days 1 through 4 concurrently with 45–50.4 Gy of external-beam radiation with the dose per fraction of 1.8 to 2 Gy for primary tumors and mediastinal lymph node regions. The clinical target volume was defined as the gross target tumor volume delineated on CT scans and other diagnostic images along with 3–5-cm cephalic and at least 5-cm caudal margins. A chest CT scan was routinely performed after chemoradiation to determine the resectability.

Surgical resections were performed using the McKeown tri-incisional esophagectomy method. The surgical approaches used were minimally invasive esophagectomy (MIE, right-sided video-assisted thoracoscopic surgery [VATS] plus laparoscopic surgery) and hybrid esophagectomy (right-sided VATS plus laparotomy). The details of surgical procedures were previously described [8]. Pathological examination that was conducted according to the 7^th^ edition AJCC TNM staging system [9]. Tumor response was graded using the CAP (College of American Pathologist) Cancer Protocol for Esophageal Carcinoma [10]. Tumor regression grade (TRG) 0 (complete response) indicated no residual cancer cells. TRG 1 (moderate response) was defined as minimal residual cancer; TRG 2 (minimal response), as partial regression of the tumor; and TRG 3 (poor response) indicated that there was no definite response identified. The presence of lymphovascular invasion (LVI), perineural invasion (PNI), and extracapsular invasion (ECI) was recorded. The presence of tumor cells at proximal or distal cut end, a circumferential margin of less than 1 mm, or M1 stage indicated incomplete resection.

Patients who were living after the operation were followed-up at our outpatient department. OS was defined as the time from the date of diagnosis until death or last known follow-up. Disease-free survival (DFS) was defined as the interval between surgical resection and the first detection of recurrence, death, or the last evaluation for recurrence. Patients who died due to surgery-related complications and those with M1 disease detected at the time of the surgery were excluded from the DFS analysis.

The Institutional Review Board of the Taipei-Veterans General Hospital approved this study and granted a waiver for the informed consent process.

### Statistical analysis

The Pearson Chi-square test was used to compare categorical variables. The Student *t*-test and ANOVA were used for comparison of continuous variables. Survival curves, plotted by the Kaplan–Meier method, were compared by the log-rank test. The Cox regression model was utilized in prognostic factor analysis. All calculations were performed using Statistical Product and Service Solutions (version 17, SPSS Inc., Chicago, IL) and a two-sided *p*-values <0.05 was considered significant.

## Results

### Patient characteristics

**Fig. 1:**
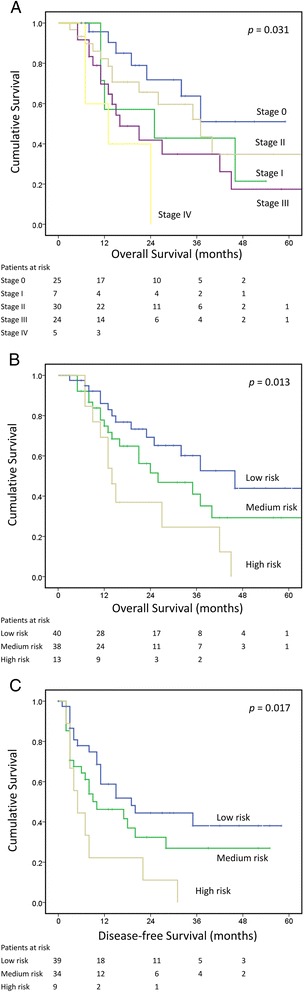
Survival curves according to AJCC staging system (**a**) and our own classification (**b** overall survival; **c** disease free survival). The curves were plotted with Kaplan-Meier method and compared with log-rank test

Table 1 shows the detailed clinical and pathological characteristics of the patients with ESCC as well as their prognostic relevance. The mean age of the patients was 55.4 +/− 9.1 years. Four patients with clinical stage T1 tumors, but associate with clinical positive nodal involvement were given chemoradiation before esophagectomy. Five patients with ypM1 stage were because of incidentally identified metastasis (lung or liver) during esophagectomy. A total of 4 patients (4.4%) died of surgery-related complications. The mean follow-up time for the entire cohort was 22.0 months (range: 3 to 67 months). The median OS was 32 months (95% confidence interval [CI], 21.9–42.1 months), with 1-year and 3-year OS rates of 78.7 and 46.5%, respectively. The median DFS was 11 months (95% CI, 4.5–17.5 months), with 1-year and 3-year DFS rates of 49.1 and 27.5%, respectively. Survival according to the AJCC staging is shown in Fig. 1a.

**Table 1:** Patient demographics and the relevance to overall survival

Variables	No. of patients	3-years survival rate (%)	Median survival, month (95% CI)	*p* value
Age (yrs)				0.144
> 55	47	53.6	42.0 (19.1–64.9)	
< =55	44	39.5	26.0 (17.2–34.8)	
Sex				
Male	88	46.3	32.0 (21.9–42.1)	0.799
Female	3	66.7	-^a^	
Location				0.136
Upper third	24	35.4	15.0 (0.0–36.6)	
Middle third	45	44.2	27.0 (13.7–40.3)	
Lower third	22	64.5	-^a^	
Differentiation				0.096
Grade I	3	33.3	12.0 (10.4–13.6)	
Grade II	42	44.1	27.0 (12.6–41.4)	
Grade III	11	37.9	11.0 (8.1–13.9)	
N/A	35	57.0	-^a^	
Tumor size (cm)				0.665
< 2.5	51	39.8	27.0 (13.4–40.6)	
> = 2.5	40	54.4	37.0 (17.3–56.7)	
cT stage				0.918
T1	4	50.0	-	
T2	17	51.5	37.0 (21.0–53.0)	
T3	66	45.7	27.0 (12.1–41.9)	
T4	4	50.0	-^a^	
cN stage				0.778
N0	27	36.5	32.0 (22.3–41.7)	
N1	24	54.8	37.0 (9.06–64.9)	
N2	17	41.0	21.0 (8.6–33.4)	
N3	13	27.2	24.0 (17.4–30.6)	
ypT stage				0.908
T0	32	53.4	37.0 (14.6–59.4)	
Tis/1	11	42.4	25.0 (6.3–43.7)	
T2	18	54.0	37.0 (15.5–58.5)	
T3	24	34.1	27.0 (14.0–40.0)	
T4	6	44.4	14.0 (2.2–25.8)	
ypN stage				0.032 †
N0	56	53.5	37.0 (21.3–52.7)	
N1	20	50.4	37.0 (2.0–72.0)	
N2	12	13.0	15.0 (12.0–18.0)	
N3	3	33.0	24.0 (0.0–49.6)	
ypM stage				0.019 †
M0	86	49.1	35.0 (23.8–46.2)	
M1	5	0.0	13.0 (0.1–25.9)	
ypStage				0.031 †
0	25	63.8	-^a^	
I	7	42.9	25.0 (0.0–58.4)	
II	30	52.2	37.0 (20.5–53.5)	
III	24	34.9	16.0 (7.5–24.5)	
IV	5	0.0	13.0 (0.1–25.9)	
TRG				0.454
0	29	57.9	37.0	
1	32	48.2	25.0 (5.8–44.3)	
2	15	17.6	27.0 (12.4–41.6)	
3	15	45.0	26.0 (3.6–48.4)	
LVI				0.024 †
No	70	55.0	37.0 (26.3–47.7)	
Yes	21	23.4	14.0 (8.9–19.1)	
PNI				0.014 †
No	76	55.2	37.0 (26.1–47.9)	
Yes	15	15.6	13.0 (9.4–16.6)	
ECI				0.002 †
No	77	50.5	37.0 (23.9–50.1)	
Yes	14	23.9	11.0 (7.9–14.1)	
Total resected lymph node number				0.134
< 18	44	55.8	37.0 (21.4–52.6)	
> =18	47	38.2	23.0 (10.6–35.4)	
Incomplete resection				0.033 †
No	63	55.0	37.0 (26.1–47.9)	
Yes	28	26.0	21.0 (9.5–32.5)	
Surgical approach				0.950
MIE	62	47.9	35.0 (22.0–48.0)	
Hybrid	29	30.7	27.0 (15.0–39.0)	
Adjuvant therapy				0.434
No	67	47.4	32.0 (18.9–45.1)	
Yes	24	42.4	35.0 (19.2–50.8)	

*CI* confidence interval, *TRG* tumor regression grade, *LVI* lymphovascular invasion, *PNI* perineural invasion, *ECI* extracapsular invasion, *MIE* minimally invasive esophagectomy and reconstruction; ^a^:median survival not reached; †: *p* < 0.05

### Prognostic value of histological factors

In the Cox regression model, we did not include M stage into the analysis because it was one of the characteristics of incomplete resection. The univariate survival analysis is shown in Table 2. Factors adversely affecting OS included LVI, PNI, ypN stage, ECI, and incomplete resection. Unfavorable prognostic factors for DFS included LVI, ECI, incomplete resection, and TRG. Since some of these factors were highly correlated (for example, TRG was significantly correlated with LVI and PNI; and ypN stage was correlated ECI), we did not perform multivariate analysis, trying to avoid the problem of multicollinearity in a multiple regression model. Of these factors, TRG, LVI, and PNI were the primary tumor features, ypN stage and ECI were the lymph node features, and resection margins were the surgical features. Patients with any of TRG2/3, LVI(+) or PNI(+) were characterized as having poor primary tumor factors, whereas patients with either ypN(+) or ECI (+) were regarded as having poor lymph node factors. Patients with incomplete resection were considered as ha ving poor surgical factors. The classification was based on the number of poor histological factors and had a range from 0 to 3 (Table 3). Using Cox regression model, we demonstrated that these factors have additional effect; which means, patients with 1, 2, and 3 poor factors had HR of 1.576, 1.728, and 3.130 compared to those without any poor factor in the overall survival analysis (*p* = 0.048). Patients with 1, 2, and 3 poor factors had HR of 1.062, 2.030, and 2.958 compared to those without any poor factor in the disease-free survival analysis (*p* = 0.027). We then classified patients with 0 poor histological factors as low risk, those with 1 or 2 factors as medium risk, and patients with 3 factors as high risk. In all, there were 40, 38, and 13 patients in the low-, medium-, and high-risk groups, respectively. The 3-year OS was 60.1, 41.0, and 24.6% in the low-, medium-, and high-risk groups, respectively. The median survival (95% CI) was 46.0 (25.7–66.3), 26.0 (8.4–43.6), and 14.0 (10.6–17.4) months in the low-, medium-, and high-risk groups, respectively (*p* = 0.013, Fig. 1b). The 3-year DFS was 38.1, 27.0, and 0.0% in low-, medium-, and high-risk groups, respectively. The median DFS survival (95% CI) was 19.0 (8.0–30.0), 10.0 (0.8–19.2), and 5.0 (2.1–7.9) months in the low-, medium-, and high-risk groups, respectively (*p* = 0.017, Fig. 1c).

**Table 2:** Prognostic factors for overall survival (OS) and disease free survival (DFS)

	OS		DFS	
	HR (95% CI)	*p* value	HR (95% CI)	*p* value
TRG (2/3 vs. 0/1)	1.455 (0.801–2.645)	0.218	1.834 (1.035–3.248)	0.038*
LVI (yes vs. no)	2.009 (1.076–3.752)	0.029*	1.975 (1.040–3.752)	0.038*
PNI (yes vs. no)	2.226 (1.144–4.331)	0.019*	1.481 (0.693–3.164)	0.311
ypN stage (+ vs. −)	2.041 (1.123–3.708)	0.019*	1.628 (0.926–2.861)	0.090
ECI (yes vs. no)	2.804 (1.404–5.599)	0.003*	2.836 (1.401–5.740)	0.003*
Incomplete resection (yes vs. no)	1.897 (1.033–3.482)	0.039*	2.254 (1.248–4.071)	0.007*

*N* = 91 and 82 for OS and DFS analysis, respectively

*HR* hazard ratio, *CI* confidence interval, *TRG* tumor regression grade, *LVI* lymphovascular invasion, *PNI* perineural invasion, *ECI* extracapsular invasion; *: *p* < 0.05 by Univariate Cox regression analysis

**Table 3:** Category of each ominous factors (primary tumor, lymph node and surgical) in our classification and the parameters in each category

Category	Tumor factor	Lymph node factor	Surgical factor
(+) if any of these parameters	TRG (2/3) LVI (+) PNI (+)	ypN stage (+) ECI (+)	Incomplete resection

### Impact of adjuvant therapy on patients with predicted poor outcomes

**Fig. 2:**
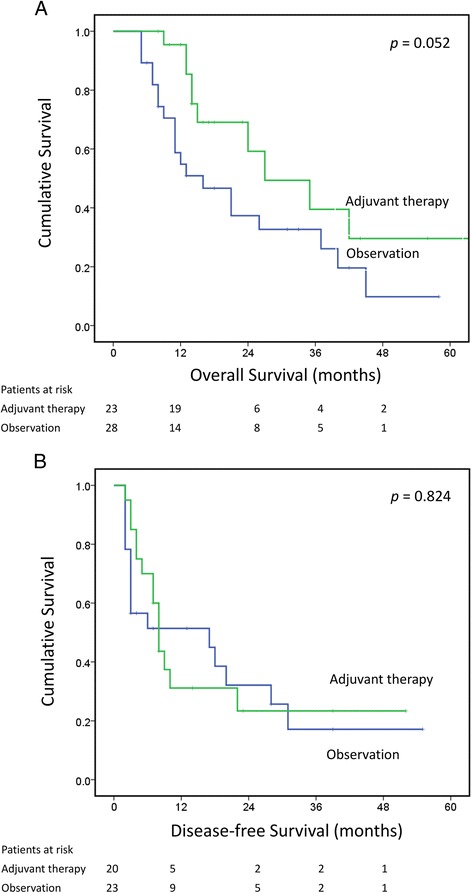
Overall survival (**a**) and disease free survival (**b**) of medium/high risk patients with (green line) or without (blue line) adjuvant therapy. The curves were plotted with Kaplan-Meier method and compared with log-rank test

We further evaluated the survival impact of adjuvant therapy in ESCC patients predicted to have worse outcomes after trimodality therapy. Of all the patients in the medium- or high-risk groups, 23 received adjuvant therapy whereas 28 received observation only. Although there seemed to be a trend towards better OS in patients receiving adjuvant therapy, no statistical significance was reached; the 1- and 3-year OS rates were 95.5 and 39.5% in patients receiving adjuvant treatment and 54.8 and 32.7% in those receiving observation only (Fig. 2a, *p* = 0.052). No significant difference was found between the two groups in terms of DFS. The 1- and 3-year DFS rates were 37.4 and 23.4% in patients receiving adjuvant treatment and 51.4 and 17.1% in those receiving observation only (Fig. 2b, *p* = 0.824). On the basis of our observations, it is suggested that adjuvant therapy did not have a significant effect on survival in patients with predicted poor outcome.

## Discussion

The current 7^th^ AJCC staging system was based on the retrospective analysis of patients who underwent surgery without induction treatment or adjuvant therapy [9]. The prognostic value of such pathologic staging for patients who received preoperative treatment is questionable [11–13]. For example, Kim et al. have shown that the staging system was not very predictive of survival in patients after preoperative treatment. In particular, there was less distinctiveness among stage subgroups. Supplementation of the AJCC TNM staging system with pathologic response for a better prediction of patients’ outcomes has been proposed for a long time [14]. Indeed, the pathological response of the tumor is a critical determinant of survival in patients receiving neoadjuvant treatment. Although chemoradiation responders have superior survival, the 5-year survival is only 18–27% in poor responders [3–6, 15, 16]. Some authors reported that non-responders to chemoradiation received no benefit and had even worse survival compared to patients treated with primary esophagectomy [4–6]. By combining classifications of primary tumor regression and lymph node status, Hölscher et al. have established a 3-grade classification with a good performance in prognostic discrimination [7]. However, Holscher`s prognostic classification was based on histological response in esophageal adenocarcinoma. Information regarding the prognostic classification of histological response in ESCC is limited in the literature. In the present study, we demonstrated the prognostic value of histological parameters including TRG, LVI, PNI, ypN stage, ECI, as well as incomplete resection, in ESCC. In addition to the well-known poor prognostic factors, such as TRG, ypN, and incomplete resection, our prognostic classification also includes LVI, PNI, as well as ECI. Schoppmann et al. have reported that both the 5-year OS (14% vs. 60%, *p* < 0.001) and the 5-year DFS (14% vs. 49%, *p* < 0.001) were significantly reduced in patients with positive LVI [17]. Chen et al. have also reported that ESCC patients with PNI-negative tumors had a 1.7-fold increase in the 5-year recurrence-free survival over the 5-year DFS for with patients with PNI-positive tumors [18]. As for ECI, D’Annoville reported that the proportion of ECI detected in N1, N2, and N3 patients was 28% (21 of 73 patients), 51% (21 of 41 patients), and 70% (17 of 24 patients), respectively. The presence of ECI seems to have negative additive impact on DFS, regardless of the pN stage [19]. ECI detected after preoperative chemoradiation and esophagectomy have been reported to be associated with a very dismal prognosis. In D’Journo’s study, the 5-year DFS rates were 46% in N0 patients, 36% in N+ with intracapsular invasion patients, and 11%, in N+ with ECI patients [20].

As the information provided by these histological factors can identify high-risk patients, the requirement of more aggressive treatment for the patients with predicted poor outcomes has to be addressed; however, the effect of adjuvant therapy in these patients is doubtful. Only a few studies have evaluated the effect of adjuvant therapy in patients who underwent trimodality treatment; this is partially because postoperative chemotherapy or chemoradiation is poorly tolerated in esophageal cancer patients. For example, 46% of patients in Stile’s study received adjuvant chemotherapy, and most of the (89%) received only one cycle [21]. In the current study, 24 patients (26.4%) received adjuvant therapy: 11 of them received adjuvant chemotherapy and 13 of them received adjuvant chemoradiation. Of all patients, 18 (75%) completed the treatment. Out of 6 cases of incomplete treatment, 5 were due to disease progression, and therefore, palliative chemotherapy was administered to the patients instead. The sixth patient had pneumonia with septic shock during the second course of chemotherapy. High completion rates of adjuvant therapy in this study may be related to the use of minimally invasive surgical techniques that resulted in earlier recovery from surgical trauma and faster return to baseline physical function, which, in turn, improved the delivery of adjuvant therapy. However, in our series, there was no significant difference in OS or DFS between patients with or without adjuvant therapy, which was compatible with the results from the literature. In the study by Meredith et al., which included 301 patients with esophageal adenocarcinoma (86.7%) and 46 patients with ESCC (13.3%), the 5-year OS and DFS were 43 and 43%, respectively, in patients who were treated with adjuvant therapy and 46 and 48%, respectively, in those who were not [6]. In their study, 34 of 262 patients (13.0%) received adjuvant therapy after preoperative treatment and esophagectomy. They found that adjuvant therapy had no impact on survival outcomes in patients who received trimodality treatment. As echoed in Meredith’s study, adjuvant therapy was not a predictor of survival in another study by Stiles et al. [21].

Our study has some limitations. First, EUS was not performed in every patient. Some patients came to us with intolerable dysphagia and near total endoscopic obstruction, which made EUS infeasible. In such cases, CT scan or bronchoscopy determined the cT3 and cT4 lesions. Second, data on tumor differentiation, which was determined by pretreatment diagnostic biopsy, was lacking for a large portion of patients. Some patients were referred to us after being diagnosed elsewhere, and we did not have an access to the biopsy specimens. Third, the small number of patients in this study limits the ability of the new classification to predict survival with significant difference between groups. Furthermore, most patients in this cohort were men, which, in agreement with the results from our nation-wide database, confirms that ESCC is the male-predominant disease in Taiwan [22]. Whether our prognostic classification can be applied to female esophageal cancer patients remains to be elucidated. Finally, although the present study is one of the few studies examining the effect of adjuvant therapy in patients who received trimodality treatment for esophageal cancer and is probably the first one discussing this topic exclusively in terms of squamous cell carcinoma histology, the sample size was small, and the assignment of patients to adjuvant therapy or observation groups was not randomized and subject to substantial bias. In general, the main indications for adjuvant therapy include positive lymph nodes, close margins, incomplete resection, and the absence of postoperative complications. We believe that further large-scale study is needed to validate our findings and determine the role of adjuvant therapy in high-risk patients.

## Conclusions

Histological factors including primary tumor factor (TRG, LVI, and PNI), lymph node factor (ypN stage and ECI), and surgical factor (incomplete resection) has a significant value in predicting survival in patients after trimodality treatments. In the future, it will be essential to establish a surveillance protocol to determine the role of adjuvant therapy in high-risk patients.

## Abbreviations

CT: Computed tomography
DFS: Disease-free survival
ECI: Extracapsular invasion
ESCC: Esophageal squamous cell carcinoma
EUS: Endoscopic ultrasound
LVI: Lymphovascular invasion
MIE: Minimally invasive esophagectomy
OS: Overall survival
PNI: Perineural invasion
TRG: Tumor regression grade
VATS: Video-assisted thoracoscopic surgery
